# Supplementary figures and images for: Effects of combined immunosuppressant and hepatitis B virus antiviral use on COVID-19 vaccination in recipients of living donor liver transplantation
Source: PeerJ. 2024 Dec 6;12:e18651. doi: 10.7717/peerj.18651 (PMC11627077; doi:10.7717/peerj.18651)

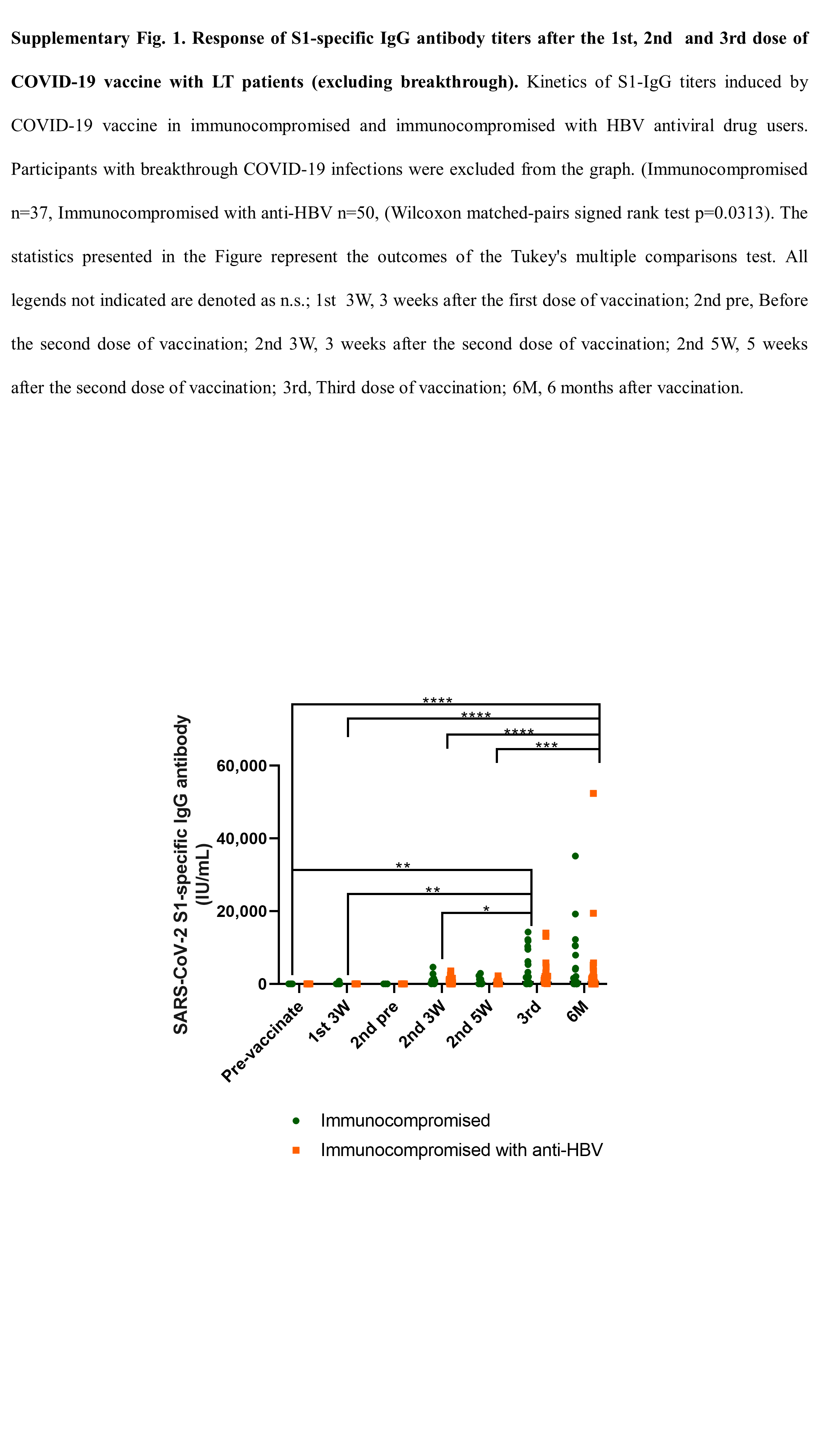

Supplement: Supplemental Information 1 — Kinetics of S1-IgG titers induced by COVID-19 vaccine in immunocompromised and immunocompromised with HBV antiviral drug users. Participants with breakthrough COVID-19 infections were excluded from the graph. (Immunocompromised n = 37, Immunocompromised with anti-HBV n = 50, (Wilcoxon matched-pairs signed rank test p = 0.0313). The statistics presented in the Figure represent the outcomes of the Tukey’s multiple comparisons test. All legends not indicated are denoted as n.s.; 1st 3W, 3 weeks after the first dose of vaccination; 2nd pre, Before the second dose of vaccination; 2nd 3W, 3 weeks after the second dose of vaccination; 2nd 5W, 5 weeks after the second dose of vaccination; 3rd, Third dose of vaccination; 6M, 6 months after vaccination. [file peerj-12-18651-s001.png]

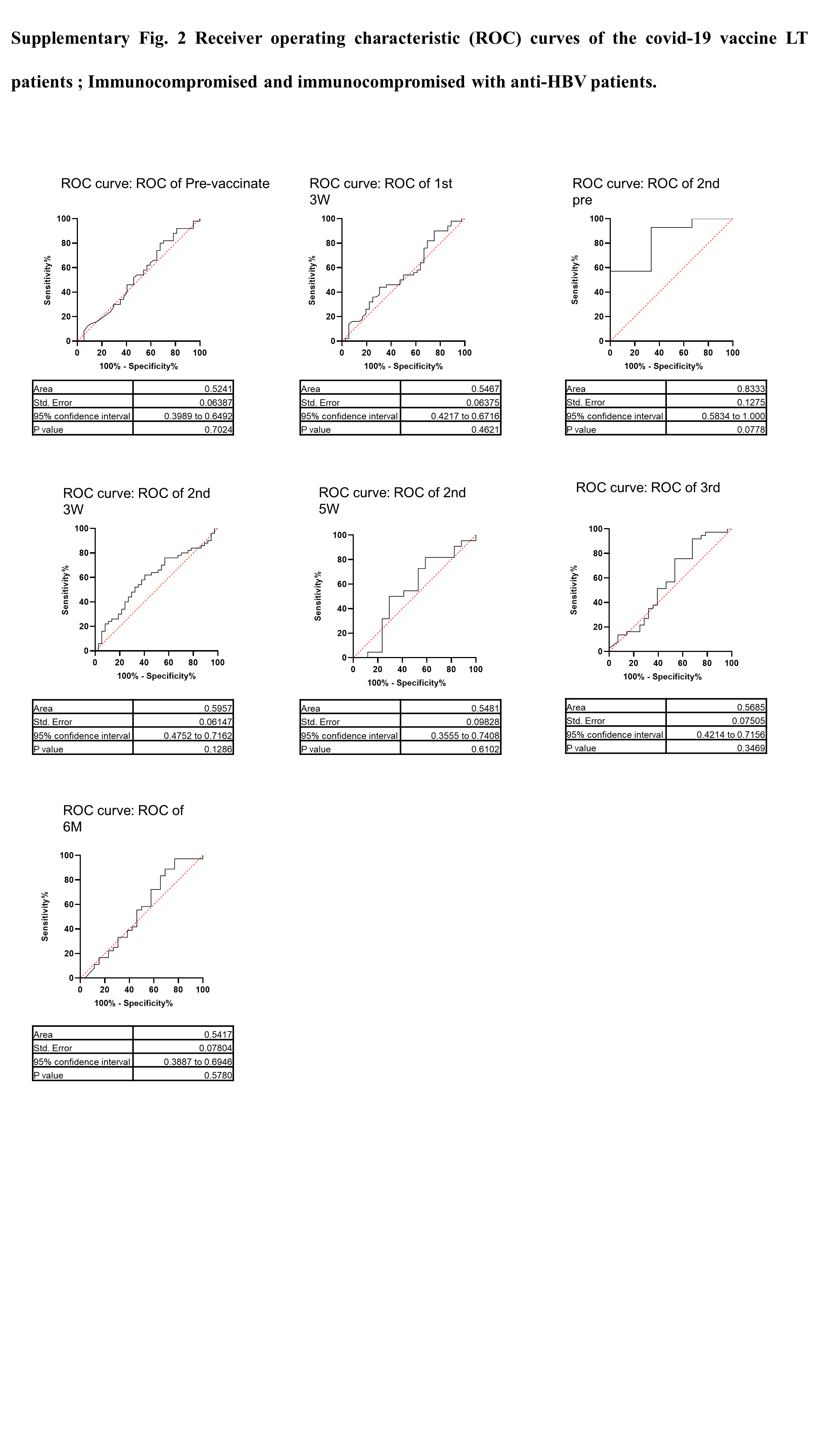

Supplement: Supplemental Information 2 — Immunocompromised and immunocompromised with anti-HBV patients. [file peerj-12-18651-s002.png]

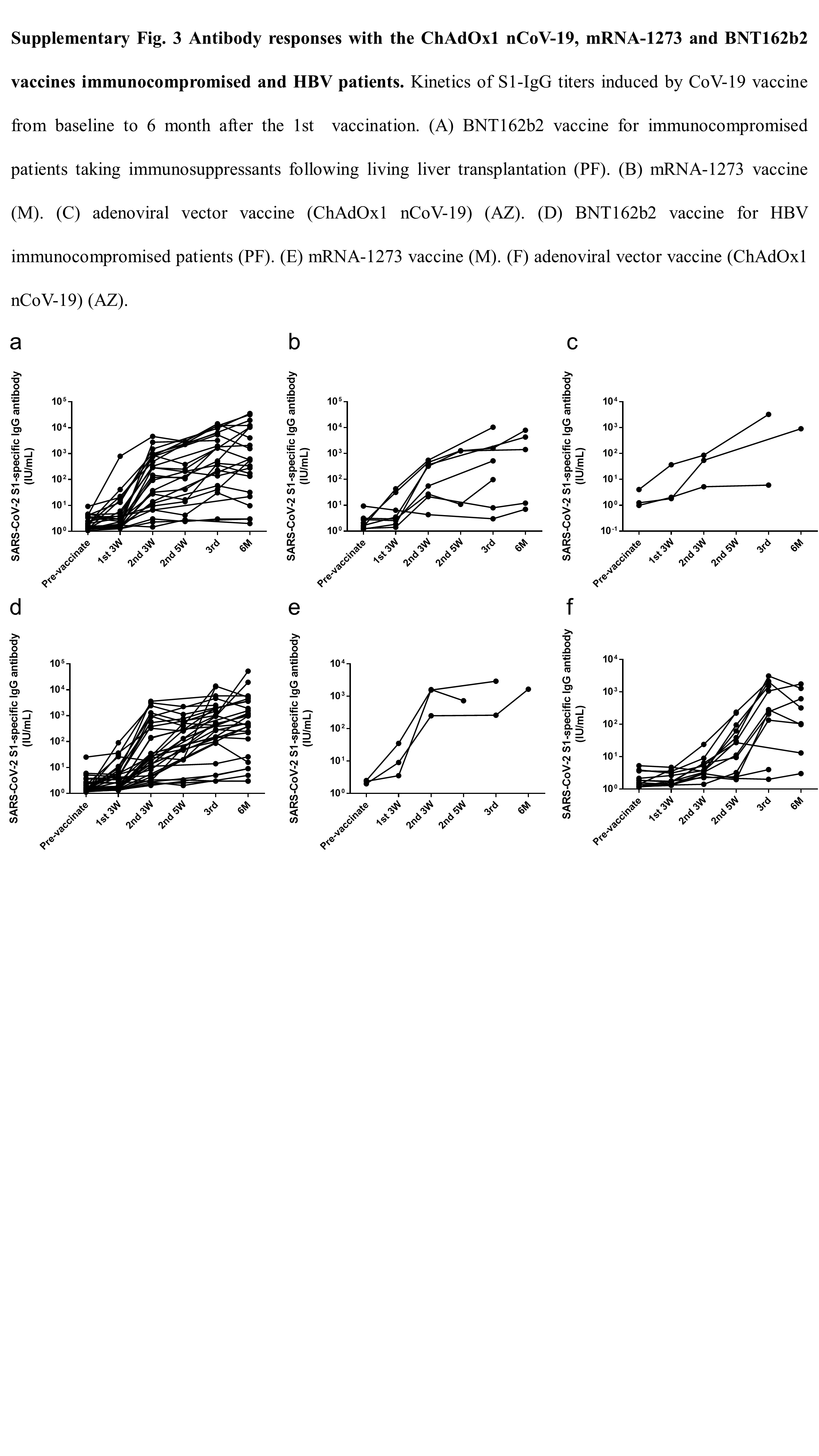

Supplement: Supplemental Information 3 — Kinetics of S1-IgG titers induced by CoV-19 vaccine from baseline to 6 month after the 1st vaccination. (A) BNT162b2 vaccine for immunocompromised patients taking immunosuppressants following living liver transplantation (PF). (B) mRNA-1273 vaccine (M). (C) adenoviral vector vaccine (ChAdOx1 nCoV-19) (AZ). (D) BNT162b2 vaccine for HBV immunocompromised patients (PF). (E) mRNA-1273 vaccine (M). (F) adenoviral vector vaccine (ChAdOx1 nCoV-19) (AZ). [file peerj-12-18651-s003.png]

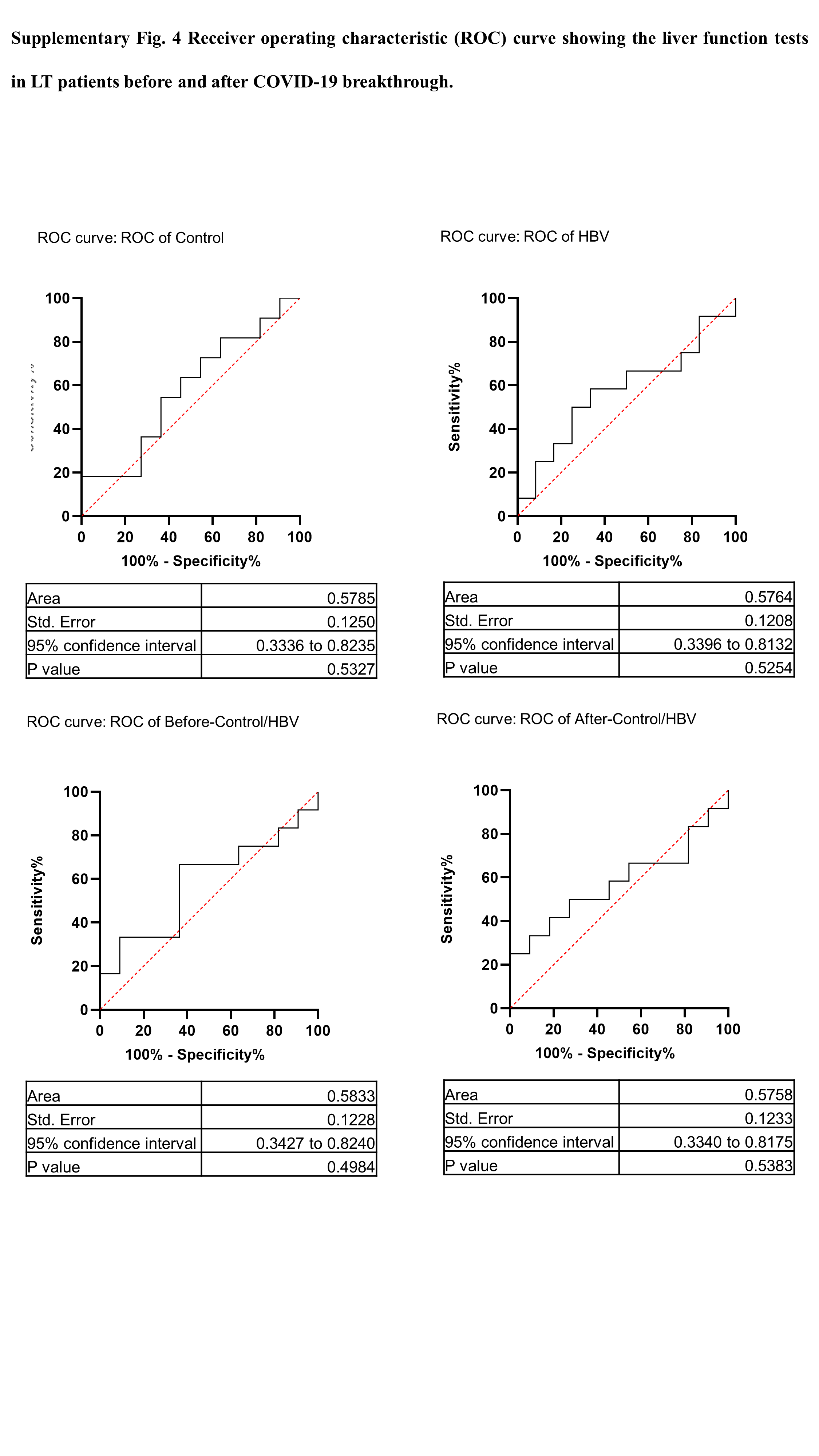

Supplement: Supplemental Information 4 [file peerj-12-18651-s004.png]
